# Evaluation of a Desktop 3D Printed Rigid Refractive-Indexed-Matched Flow Phantom for PIV Measurements on Cerebral Aneurysms

**DOI:** 10.1007/s13239-019-00444-z

**Published:** 2019-12-09

**Authors:** W. H. Ho, I. J. Tshimanga, M. N. Ngoepe, M. C. Jermy, P. H. Geoghegan

**Affiliations:** 1 Department of Mechanical and Industrial Engineering, University of South Africa, Johannesburg, South Africa; 2 School of Mechanical Aerospace and Industrial Engineering, University of the Witwatersrand, Johannesburg, South Africa; 3 Department of Mechanical Engineering, University of Cape Town, Cape Town, South Africa; 4 Department of Mechanical Engineering, University of Canterbury, Christchurch, New Zealand; 5 Biomedical Engineering, School of Life and Health Sciences, Aston University, Birmingham, England

**Keywords:** Cerebral aneurysm, Particle image velocimetry, Flow phantom, PIV, 3D printing, Additive manufacturing, Refractive-matched, Experimental fluid dynamics, *In vitro* experimentation, Bio-fluids, Haemodynamics

## Abstract

**Purpose:**

Fabrication of a suitable flow model or phantom is critical to the study of biomedical fluid dynamics using optical flow visualization and measurement methods. The main difficulties arise from the optical properties of the model material, accuracy of the geometry and ease of fabrication.

**Methods:**

Conventionally an investment casting method has been used, but recently advancements in additive manufacturing techniques such as 3D printing have allowed the flow model to be printed directly with minimal post-processing steps. This study presents results of an investigation into the feasibility of fabrication of such models suitable for particle image velocimetry (PIV) using a common 3D printing Stereolithography process and photopolymer resin.

**Results:**

An idealised geometry of a cerebral aneurysm was printed to demonstrate its applicability for PIV experimentation. The material was shown to have a refractive index of 1.51, which can be refractive matched with a mixture of de-ionised water with ammonium thiocyanate (NH_4_SCN). The images were of a quality that after applying common PIV pre-processing techniques and a PIV cross-correlation algorithm, the results produced were consistent within the aneurysm when compared to previous studies.

**Conclusions:**

This study presents an alternative low-cost option for 3D printing of a flow phantom suitable for flow visualization simulations. The use of 3D printed flow phantoms reduces the complexity, time and effort required compared to conventional investment casting methods by removing the necessity of a multi-part process required with investment casting techniques.

## Introduction

An aneurysm is a balloon-like bulge that forms on a blood vessel as a result of weakened vessel wall layers. The most dangerous aneurysms are found in the Circle of Willis and on the abdominal aorta. For cerebral aneurysms, rupture risk is 0.6–1.3% per annum.^45^ The main challenge is that when rupture occurs, there is subsequent morbidity or mortality. Cerebral aneurysms can be treated by surgical or endovascular techniques, such as coils, flow diverters and stents. One of the main challenges relating to treatment is that advances in medical imaging technology have led to an increase in accidental sighting of un-ruptured aneurysms during routine scans for other conditions.^39^,^42^,^46^ Given the patient-specific nature of the disease, clinicians are presented with a dilemma as the decision to treat an aneurysm, which would have remained asymptomatic exposes the individual to iatrogenic risk. Conversely, the decision not to treat could later prove fatal should the aneurysm rupture.

Fluid dynamics is important in the investigation of the flow characteristics within an aneurysm and in cardiovascular diseases in general.^7^,^10^,^20^,^21^,^24^–^28^,^33^,^35^,^36^ Various computational tools have been developed in an effort to predict aneurysm outcome on a per patient basis.^5^,^19^,^33^,^34^ Some of these have focused solely on the haemodynamics, while others incorporate aneurysm-associated thrombosis.^8^,^30^–^32^,^37^ An essential component in the credibility of these tools and models is validation with experimental data. Ideally, *in vivo* data would be the most physiologically representative means of validating computational models, however, observing phenomena like long-term thrombosis proves challenging. *In vitro* tools which can be used to generate data for validation are desirable as there can be greater control over the experimental process.^16^ To date, there have been a large number of *in-vitro* studies of haemodynamics.^2^–^4^,^11^–^13^,^17^,^22^,^40^,^41^ For both *in silico* and *in vitro* setups, the reconstruction of patient-specific anatomical models is beneficial. These are often derived from computed tomography (CT) or magnetic resonance images (MRI).

Particle image velocimetry (PIV) is an *in vitro* fluid dynamic measurement method that is commonly used for external and internal flow studies and is particularly useful for bio-fluid flow problems, such as flow in aneurysms.^9^,^13^,^16^,^29^,^38^ Aside from vascular flow, it has also been employed for the investigation of respiratory studies.^14^ The majority of flows of medical interest occur in highly complex internal channels. In order to utilize PIV to measure bio-fluid flows, flow phantoms have to be constructed from an optically transparent material. To date, the most common method of constructing flow phantoms employs an investment casting method using transparent grade silicone and a 3D printed (or other forms of rapid prototyping) core. The process involves fabricating the core, polishing the core, casting a negative of the core using silicone and lastly removing the core.^15^,^18^,^47^ This process is both laborious and time consuming. Each stage of manufacture compounds the geometric error caused by the manufacture process. On average it takes about two weeks to manufacture a phantom. There are also several pitfalls at each stage of the process that if they occur, will require the whole construction to be restarted from the beginning. In addition, there is a risk that air bubbles could be trapped during the casting process, making the flow phantom unusable if the bubbles are significantly large and in the field of view. Therefore the possibility of manufacturing a phantom in a one stage process would vastly improve its quality.

Recent advances in 3D printing, particularly in the expanded range of materials and resolution of the prints have made it possible to directly print the *in vitro* flow phantoms out of transparent resin. There are two main types of 3D printing techniques that are commonly available commercially and can print optically clear resins. They are PolyJet and Stereolithography (SLA). Both uses a liquid resin but PolyJet prints in air in a raster pattern of straight lines whilst SLA prints in a liquid bath. This difference may have an impact on the clarity of the eventual printed model. Further discussion on this is given later in this paper. Different types of method-resin(s) combinations (from various manufacturers) are available on the market that are suitable for use in direct printing of optically clear flow phantoms, such as:

1. PolyJet with Veroclear resin from Stratasys
2. Stereolithography (SLA) with FLGPCL series (FLGPCL*XX*) resins from Formlabs
3. SLA with ClearVue from 3D systems
4. VisionClear resin from Luxexcel

One of the challenges of using 3D printed flow phantoms is in the matching of the refractive index (RI), due to the high refractive indices of transparent polymers compared with cast silicone. This limits the possible working fluids suitable for use. Recently Aycock *et al*.^1^ reported the successful use of Polyjet printed Veroclear material to directly 3D print a flow phantom of an anatomical vascular model. The working fluid used was a mixture of 53% sodium iodide, 26% glycerol and 21% distilled water (by mass). They also reported differences in the optical transparency with print orientation. In this study, we investigate the direct printing of flow phantoms from a less expensive material (FLGPCL*XX* resin from Formlabs), printed with the SLA technique on a desktop Form 2 printer. In addition to being less expensive, SLA could potentially create smoother internal surfaces because of the lack of requirements for support material.^23^ We demonstrate the process with an aneurysm model, but it can be applied to any internal flow applications.

## Materials and Methods

### Fabrication of the Flow Phantom

**Figure 1:**
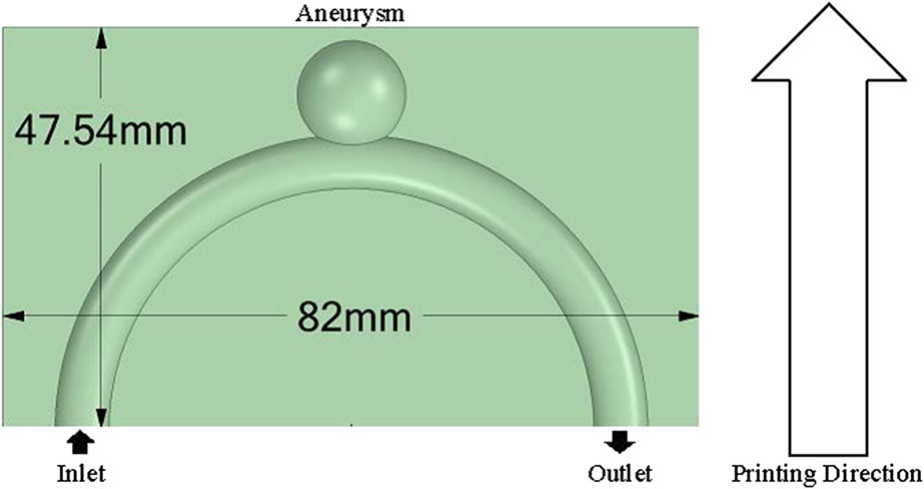
Idealised model of a cerebral aneurysm, which is modelled as a sphere.

The flow phantom was printed on a Formlabs Form 2 desktop SLA printer using clear photopolymer resin FLGPCL*XX*. An idealised geometry of a cerebral aneurysm was adapted from the model developed by Mulder *et al*.^29^ The geometry was created using CFD-GEOM (ESI Group, Paris, France), converted to a discrete surface and then saved as a stereo-lithography (STL) file. The model consists of an inlet and an outlet, as illustrated in Fig. 1. The aneurysm is modelled as a sphere and the neck of the aneurysm is located at the intersection between the sphere and the vessel.

**Figure 2:**
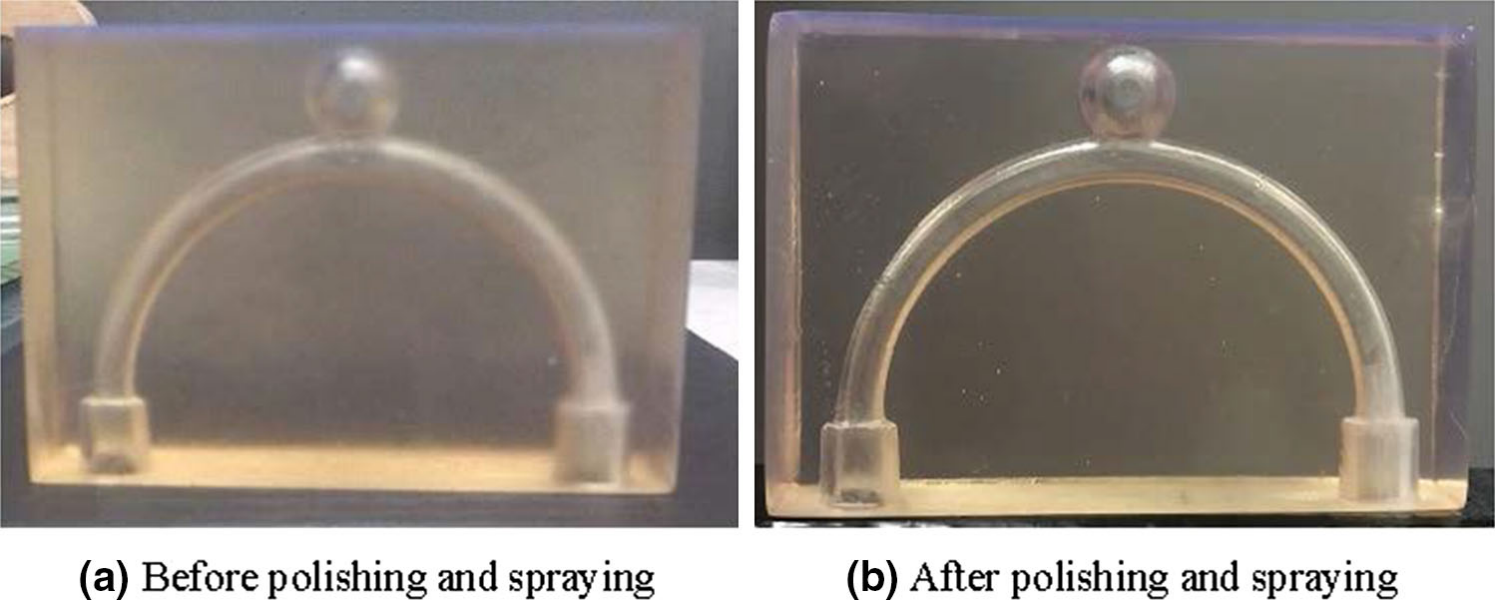
3D printed flow phantom of idealised geometry before (**a**) and after (**b**) polishing and spraying.

The printing process starts with a 3D Design of the model using computer-aided-design (CAD) software. Printing was performed at the finest resolution achievable which was 25 *µ*m. The completion of the printing process is followed by cleaning in isopropyl alcohol (IPA) to remove uncured resin from the print surfaces. The model is then placed in sunlight for at least an hour in a post-curing process as recommended by the printer and resin manufacturer. No specific control of temperature was employed. Finally the model is finished by sanding and spraying of the outer planar surfaces. Sanding was performed using sandpaper of reducing grit size, with a final grit of P2000. The final process was the manual spraying of the external surfaces with a clear fast drying aerosol lacquer based spray paint (Spraymate Clear), which provides a drastic improvement to the final clarity. A picture of the models (pre-polishing and spraying vs. post-polishing and spraying) is shown in Fig. 2.

**Figure 3:**
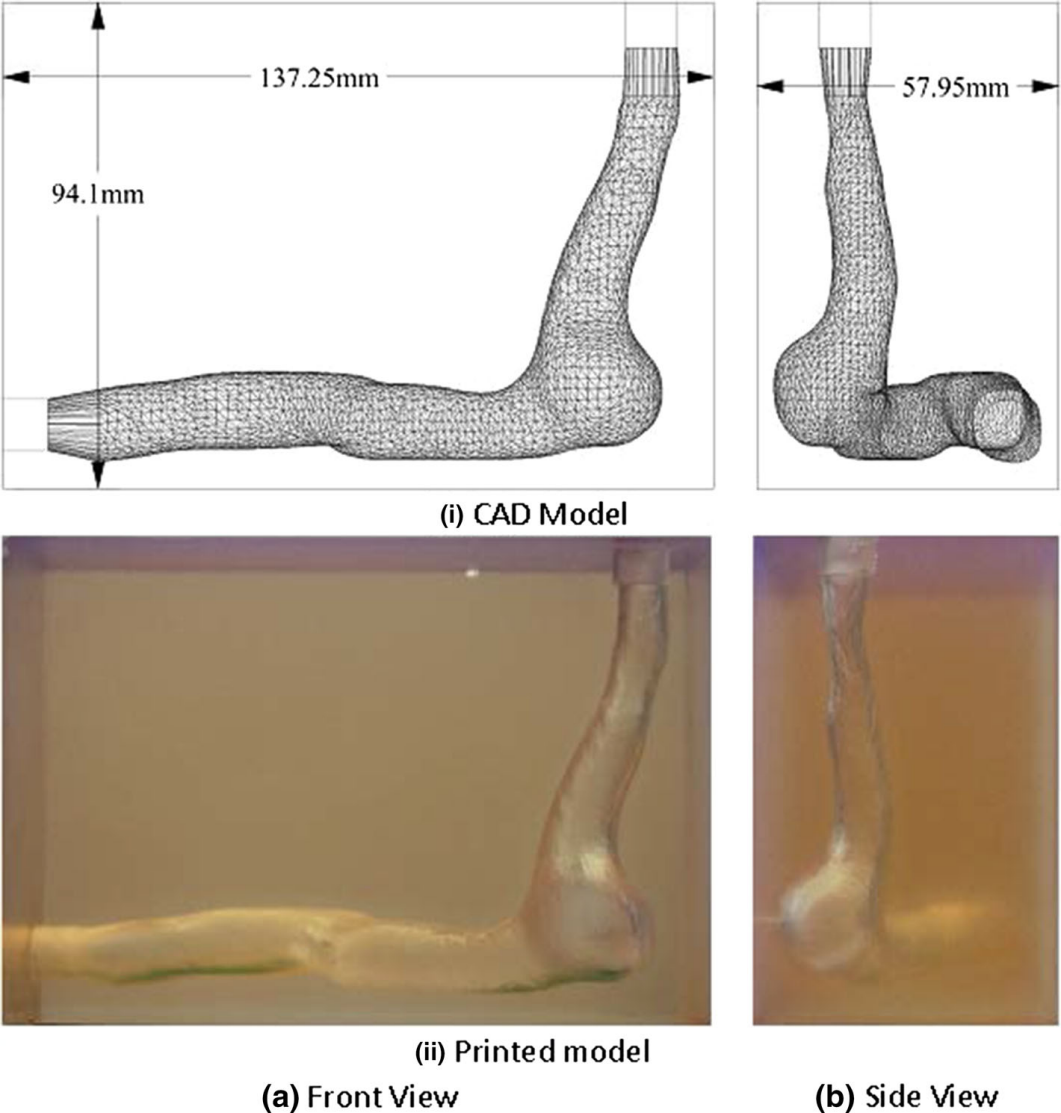
CAD (i) and actual printed model (ii) of a physiologically realistic geometry.

To further demonstrate the clarity of the material, a patient-specific model with highly complex geometrical features was printed and is shown in Fig. 3. The size of this model is fairly large compared with that of the idealised model.

### Refractive Index of the Material

The refractive index (RI) of the printed material was measured with a Bellingham and Stanley Abbé refractometer and a white LED light source. Measurements were made on flat facets of approximately 12 mm × 35 mm cuboid samples printed especially for the RI measurement. The samples were coupled optically to the refractometer prism with a thin film of bromonaphthalene. The refractometer was calibrated with deionized water and a glass slide of known RI of 1.51. The uncertainty in RI ranged from ± 0.001 to ± 0.01 with the larger values resulting from uneven sample surfaces blurring the extinction angle.

The measured RI of the printed FLGPCL*XX* resin was 1.507 ± 0.003. Aycock *et al*. did not report the RI of Veroclear hence a measurement was performed using the same technique. The measured RI was 1.51 ± 0.01. Cast silicones can have lower RI.^18^,^40^

At such high RI, the common working fluid used in cast silicones models (water-glycerol mixture) could not be used. Jermy compiled a list of suitable working fluids with zinc iodide, sodium iodide and ammonium thiocyanate shortlisted as potential candidates with the highest RI amongst those listed.^19^ A fluid composed of 66% ammonium thiocyanate (NH_4_SCN) and 34% de-ionised water at room temperature was eventually used as the working fluid that could match the RI of the model. NH_4_SCN is endothermic when mixed with water, and it should be allowed sufficient time to heat up before use. Some NH_4_SCN may be replaced with glycerol, to reduce the cost if required.

**Figure 4:**
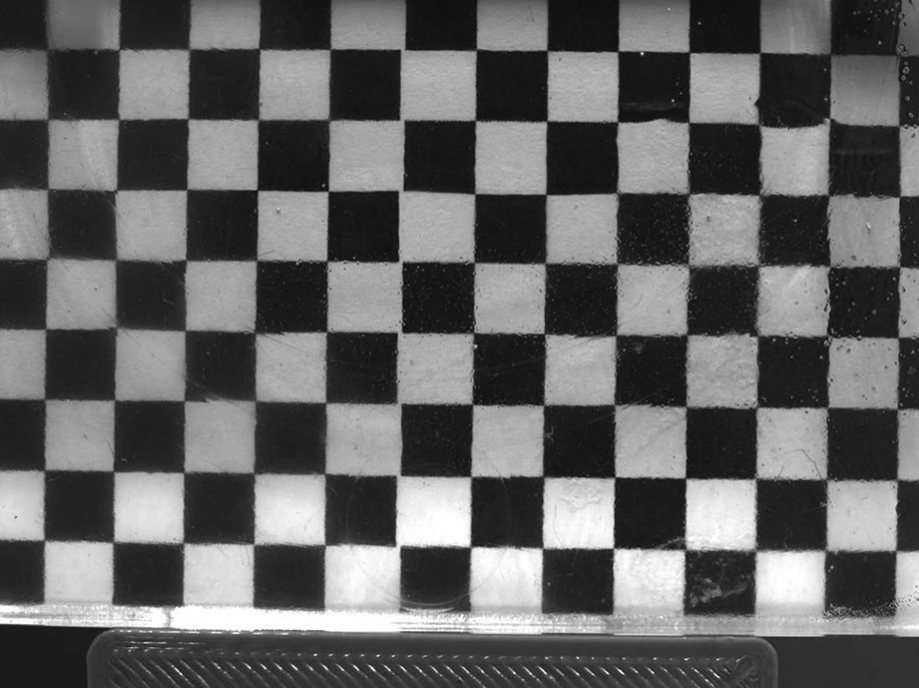
Matching of refractive index to printed phantom.

Visual inspection to detect any distortion due to mismatch of RI was performed with a checkered grid placed behind the idealised flow phantom (Fig. 4). Although this does not prove a comprehensive matching of the RI, it does indicate that with careful and appropriate manipulation of the constituents, a working fluid with RI sufficiently close to that of the model can be achieved.

### Viscosity and Density of the Working Fluid

**Figure 5:**
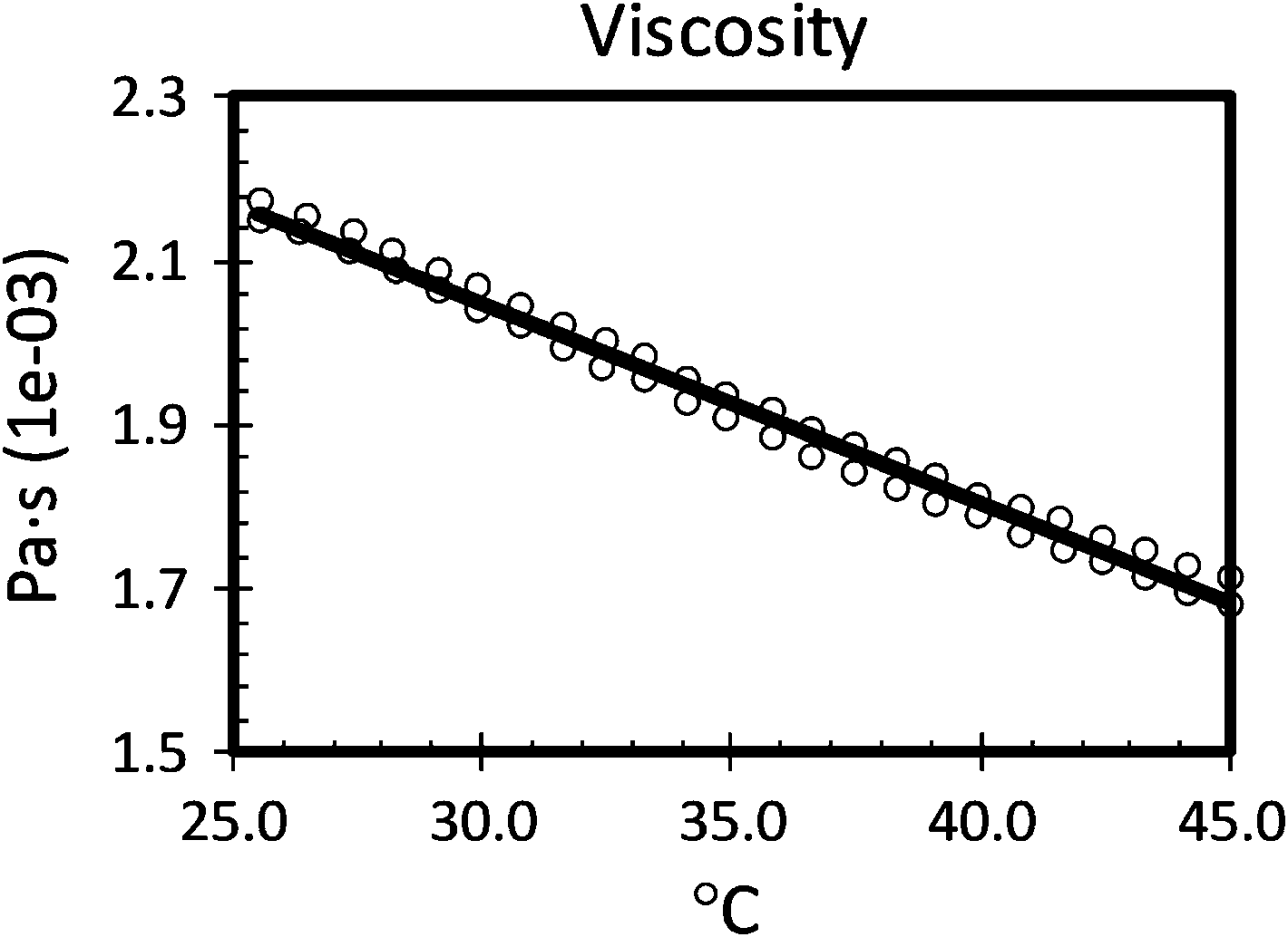
Viscosity measurements of the working fluid.

The viscosity of the working fluid used for this investigation is given in Fig. 5. The viscosity of the working fluid was measured using a TA instruments discovery hybrid rheometer. A steel Peltier parallel plate of size 40 mm was used and a temperature sweep was performed, beginning with 25 °C and ending at 45 °C. A shear rate of 200 s^−1^ was applied.

The experiment was repeated and two sets of measurements were taken and the line is the best-fit line from both sets of data combined.

The viscosity of the working fluid is not exceptionally high thus allowing for the selection of easily available pumps and fittings for the flow set-up. In addition, the relative density of the fluid 1.131 at 23.7 °C which matches quite closely with the density of commonly available seeding particles thus reducing the effects of buoyancy of the particles during low flow applications.

Although the working fluid’s viscosity and density are distinct from blood, this can be overcome by appropriate matching of Reynolds and Wommersley numbers to achieve dynamic similarity.

### Surface Roughness Measurements

Roughness of the surface in contact with the fluid is a very important parameter when conducting fluid dynamic experiments. Rougher surfaces induce laminar to turbulent transition early and affect both the bulk flow dynamics as well as the local wall shear stress (WSS) which is an important consideration in hemodynamic studies. Most previous PIV experiments on vascular flows using the conventional investment casting method only qualitatively describes the smoothness of the internal flow channel as being “smooth to touch”.

To measure the surface roughness of the internal flow channel, a cuboid solid print of the same material (but an opaque version) was used and the surfaces were scanned using the SmartZoom 5 Digital Microscope from Carl Zeiss. The sample was not polished or sprayed so that it will be similar to the internal surfaces of the flow phantoms where polishing and spraying is challenging. The opaque version of the material was used to enhance the optical measurements of the microscope. Both horizontal and vertical surfaces were scanned to check if there were differences in roughness on surfaces parallel and perpendicular to the print direction. Scanning on both surfaces was performed at different locations to get a more representative gauge of the roughness. The on-board software of the SmartZoom 5 Digital Microscope was able to provide visuals but not calculate roughness values directly. Hence a visual estimate of the maximum height of the profile Rt was performed by adjusting parallel measurement lines on the software GUI. Horizontal lines were placed (within one-pixel accuracy) at the maximum valley depth (Rv) and maximum peak height (Rp) and Rt calculated as the difference

The arithmetic average roughness Ra for the horizontal and vertical surfaces was calculated by the values extracted from the software for the entire surface and are 2764 and 5093 nm respectively. The Ra value for the vertical surfaces are higher than that for the horizontal surfaces which is expected as they cut across the printing layers. The Rt values corresponds to a Roughness N ISO Grade Number between N6 and N8. The Ra values corresponds to between N8 and N9.

### Minimum Resolution of Printed Channels

**Figure 6:**
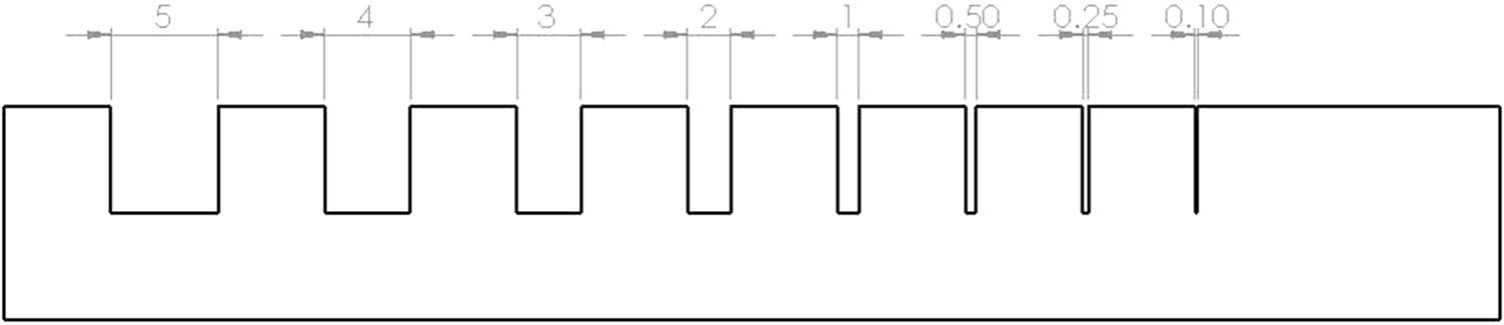
Channel width test sample (all dimensions in mm).

To assess the accuracy of the printer at printing channels in the chosen material, a separate sample was produced with descending channel size from 5 to 0.1 mm (Fig. 6). An image of the channel was then captured with an IDS UI-3130CP-M-GL Rev.2 800 × 600 pixel camera with a Fujifilm 25 mm lens. The image was manually measured using the in-built image viewer function in MathWorks MATLAB R2016a with a resolution of 13.7 pixel/mm. There were six measurements taken for each channel with the final results in Table 1. It should be noted that channel sizes of 0.5, 0.25 and 0.1 mm were not resolvable with the printer accuracy and they could not be produced in the final print. This difference between the resolution setting of the printing process and the printed model could be due to insufficient time for curing of each “layer” during the printing process hence leading to a “melting” effect. This is however not a setting that can be altered on the user-interface of the printer.

**Table 1:** Design vs. measured channel width.

Designed channel size in mm	Measured channel size in mm (standard deviation)
5.00	4.85 (0.021)
4.00	3.93 (0.035)
3.00	2.95 (0.038)
2.00	1.81 (0.022)
1.00	0.86 (0.030)

### Steady-State PIV Measurement

In order to demonstrate the applicability of the flow phantom for PIV, a planar PIV experiment was set-up with a representative steady flow rate through the idealised flow phantom.

The flow set up consists of a reservoir, a header tank with an overflow and a mini turbine flow meter. A submersible pump was used to pump the working fluid from the reservoir to the header tank located at an elevated height where the working fluid is being fed by gravity through a mini turbine flow meter and then through the flow phantom and back to the reservoir again. The header tank had an overflow, which maintained the flow rate at a constant value.

The working fluid was seeded with 10 *μ*m borosilicate glass particles and PIV images were captured using a PCO pixelfly USB 14 bit CCD PIV camera with the flow phantom being illuminated by a Firefly PIV and Imaging Laser from Oxford lasers.

Flow rate in the experiment was appropriately set to achieve dynamic similarity with *in-vivo* conditions by matching the Reynolds number at 106. This simulated the upper limit of the flow rate expected through a cerebral aneurysm and corresponds to an average shear rate more than 100 s^−1^ within the blood in *in-vivo* conditions. At this high shear rate, the viscosity of blood is constant.

As the focus of this paper is on demonstrating the applicability of the 3D printed flow phantom for use in PIV experiments, cross-correlations were made only in the idealised model although a physiologically accurate model was fabricated and PIV images taken.

## Results

### Captured PIV Images

**Figure 7:**
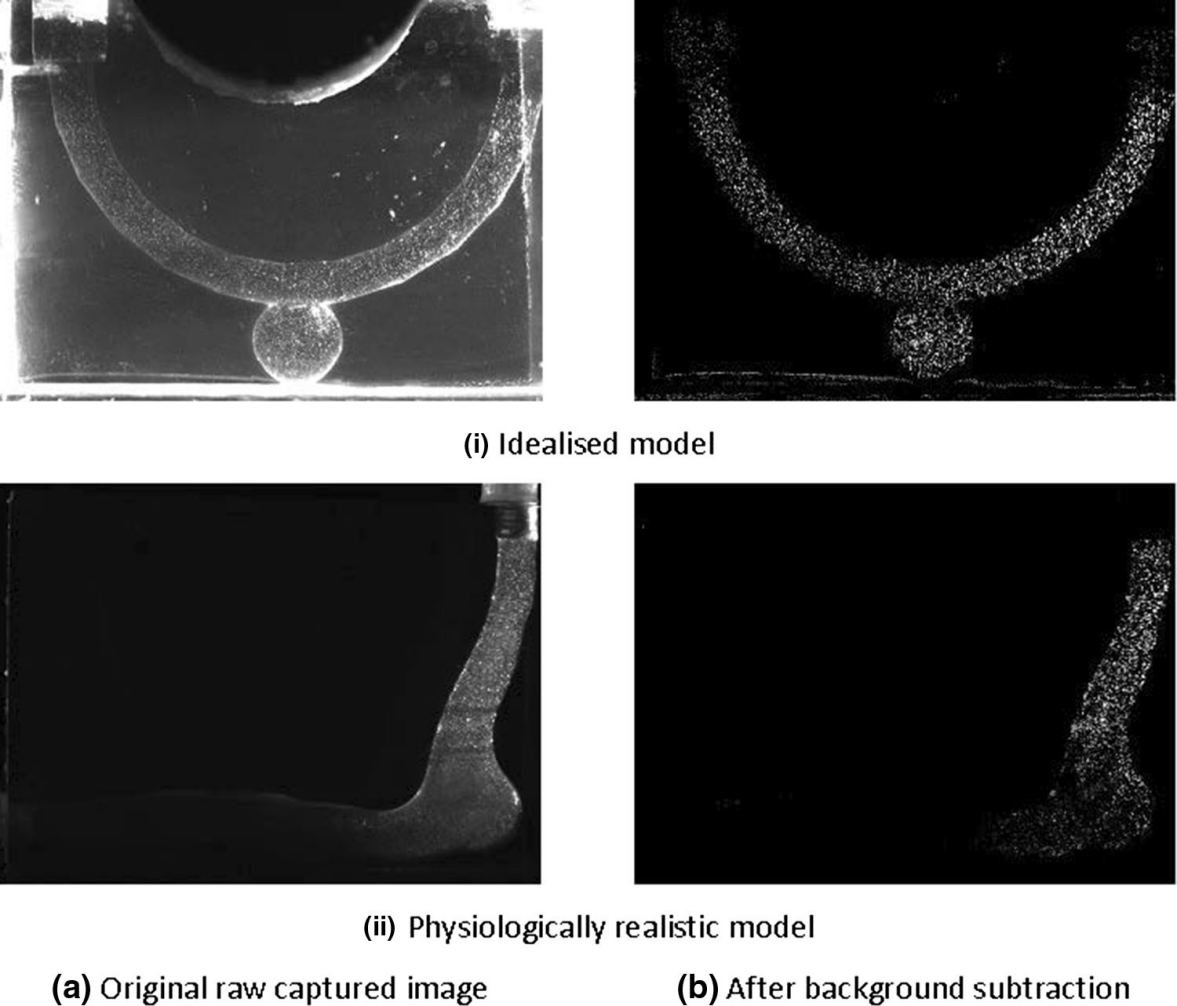
PIV images of the idealised and physiologically realistic models at various stages of the pre cross-correlation process.

**Figure 8:**
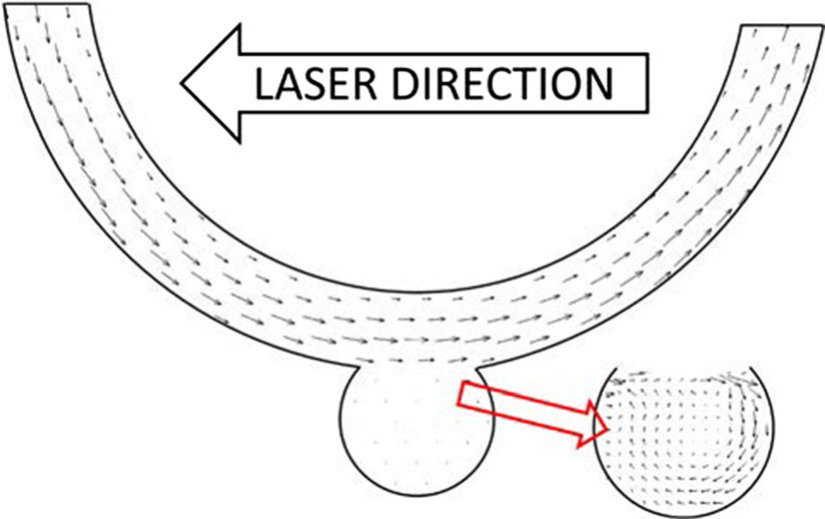
Flow vectors for idealised model.

Images used for PIV analysis are presented in Fig. 7. These images provide an indication of the “quality” that can be achieved with this printed flow phantom. These include the raw image (i) after background subtraction is performed with brightness increased uniformly for better contrast (ii) and a sample of the processed results (Fig. 8).

Processing of the results for the idealised model was performed using the open-source MATLAB code PIVlab 1.4.1^43^,^44^ and post-processing performed using ParaView. The idealised flow model was orientated with the aneurysm at the base due to the constraints with the experimental rig.

The images are sufficiently clear with the particles easily distinguishable, especially after background subtraction processing. The cross-correlation code PIVlab is able to produce appropriate results clearly showing the flow recirculation about an axis that is off-centre within the aneurysm. This is consistent with the previous study by Hoi *et al*.^17^

However, it is noted that there is an obvious observable difference in brightness between the “leading” and “trailing” portion of the idealised model from the point of view of the laser direction due to attenuation of the light sheet. This may cause difficulties when using a large model, but is appropriate for the current set-up. Note that the brightness difference in the physiologically realistic model is not due to the attenuation but due to the certain parts of the flow channel not being in the same plane.

## Discussion

A Formlabs Form 2 Desktop SLA printer printing FLGPCL*XX* clear photopolymer resin can produce a flow phantom that compares well with the option of using a Stratasys PolyJet printer with Veroclear. Both the printer and the raw resin are significantly lower in cost when compared to the Veroclear option. Aycock *et al*.^1^ did not report roughness measurements, hence no comparison was possible but SLA printed models potentially could produce smoother interior channels due to the lack of need for support material. The FLGPCL*XX* model had a Ra of 2.764 and 5.093 *μ*m when measured aligned with and across the layers using an optical microscope. The Rt values corresponds to a roughness N ISO Grade Number between N6 and N8 and are acceptable for the current application. In comparison, it is still higher than cast silicone (1.27 *μ*m) but better than two other 3D printed materials (8.87 and 16.07 *μ*m) as reported by Cloonan *et al*.^6^ A solution of 66% NH_4_SCN powder dissolved in 34% de-ionised water (by weight) produces a working fluid of comparable RI as the printed model and no observable distortions in grid lines were present when a checkered grid was placed behind the flow phantom. The resulting solution is compatible with glycerol, which could be used to further “control and refine” the RI of the working fluid. In addition, the current material does not exhibit the blurring of the seeding particles due to different print orientations as reported by Aycock *et al*.^1^

In that study, limitations in-terms of clarity of the particles was found when using 3D flow models printed using the Polyjet technique with different print orientations even though the clarity and transparency is acceptable for PIV and the RI between the working fluid and printed material matched. Little explanation was provided as to why this happens. Our work made use of 3D printed flow models using the Stereolithographic (SLA) technique. Several prints were made and observations shows no loss of PIV image quality in different print orientations. A possible explanation to this phenomenon could be due to differences in how the two techniques work.

Polyjet 3D printing uses a liquid resin that cures with exposure to air and the print head travels in a raster pattern of straight lines. It (as with FDM) prints in air whereas SLA, prints by curing the material in a liquid bath of the resin and successive layers are polymerized beneath each surface, resulting in a homogenous final material. Printing using the Polyjet method could lead to either alternating strips of cured resin material sandwiching rows of air (which does not happen in the SLA process as the entire operation is being performed submerged in the liquid) or the resin in each new printed line fusing with the side of a previous line (which has been exposed to air and has aged slightly) which may give a slight modulation in the RI of the cured polymer.

**Figure 9:**
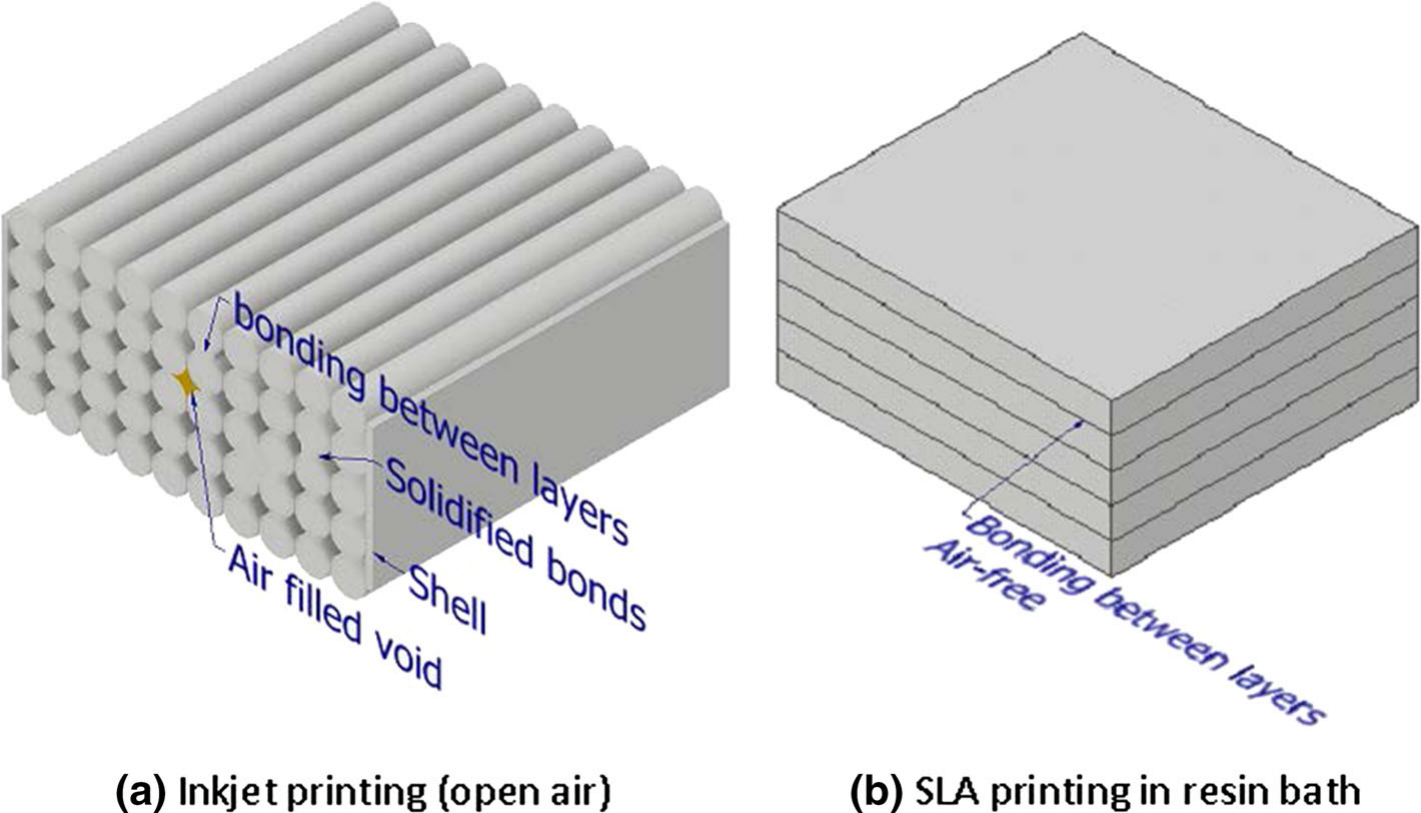
Idealised representation of the final print material between (**a**) Polyjet and (**b**) SLA processes.

An idealised illustration of the material printed using both processes highlighting the difference in the microstructure is given in Fig. 9. Grey indicates the printed material sandwiching either air or a material with different optical properties as previously described.

When conducting PIV experiments, light from the laser, or light scattered from the particles, strikes the flow phantom and depending on the position of the laser and camera with respect to the “printed material lines”, may result in modulation of the light intensity giving the smudged streaks and “spreading of the laser light sheet intensity” described by Aycock *et al*.^1^ If this effect is true, it will be more prominent the higher the resolution of the print as with a narrower gap, light will tend to enter the “air void” or “new material” with a larger angle-of-incidence.

Although there are many advantages to this method of fabricating the flow phantom, the high RI of the resin, (measured as 1.507 ± 0.003) however, makes common fluids used for similar PIV experiments on cast silicone such as glycerol-water mixture unsuitable and salt solutions have to be employed. In comparison, the measured RI for Veroclear was 1.51 ± 0.01 and VisionClear from Luxexcel (another potential material for this application) is reported to have an RI of 1.53. The Luxexcel printer does not seem to be table-top size. Cast silicone has a lower RI of between 1.41 and 1.47.^19^

In addition, the current method is not feasible for printing channels with resolution below 1 mm but has an error of not more than 0.2 mm difference from the original CAD model. This could pose problems when printing geometries with very intricate “spacings” but would be appropriate for other models. Users are recommended to take these inaccuracies into account when choosing to use this method to fabricate flow phantoms. Finally, the attenuation of light through the model may be a concern when very large models are required.

In conclusion, this study builds on the work of Aycock *et al*.^1^ and presents an alternative low-cost option for 3D printing of a flow phantom suitable for PIV or other flow visualization techniques to study internal flows. The use of 3D printed flow phantoms reduces the complexity, time and effort required compared to conventional investment casting methods. It offers further advantages when used to fabricate models with complex geometries and narrow channels as the removal of the “male” mould material after casting can pose difficulties. Although there are new challenges emanating primarily from the optical properties of the material, it has been demonstrated that photopolymer FLGPCL*XX* from Formlabs is suitable for this and similar applications.
